# Interpretation challenge of small copy number variations in the imprinting regions

**DOI:** 10.1002/mgg3.1961

**Published:** 2022-04-28

**Authors:** Yi Ning, Megan Czekalski, Sylvia Herrada, Carol Greene

**Affiliations:** ^1^ Department of Pathology University of Maryland School of Medicine Baltimore Maryland USA; ^2^ Department of Pediatrics University of Maryland School of Medicine Baltimore Maryland USA

## Abstract

We report the findings of small CNVs in two newborns in the genomic imprinting regions. They exemplified the challenge of interpreting small CNVs in diagnostic samples. Careful detection of small CNVs in the imprinting regions and effective genetic counseling are of clinical and reproductive significance

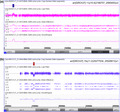

To the editor,

1

Chromosomal microarray is a powerful technology for the detection of genomic imbalances. It has become the first‐tier clinical diagnostic test for patients with developmental disabilities or congenital anomalies (Miller et al., 2010). The American College of Medical Genetics and Genomics (ACMG) and the Clinical Genome Resource (ClinGen) have established standards and recommendations for the interpretation and reporting of constitutional copy number variants (CNVs) detected by the chromosomal microarray (Riggs et al., 2020). ClinGen has collected phenotypic and clinical information on variants across the genome and developed consensus approaches to identifying their clinical significance. The ClinGen database has been updated continuously.

While chromosomal microarray has been increasingly used in the diagnostic laboratories, interpretation of small CNVs can be challenging. Recently, we observed two small CNVs in two newborns. According to the ClinGen dosage sensitivity map for the corresponding regions, there are no known dosage sensitivity genes in these regions. However, both CNVs are in the known genomic imprinting regions, which triggered further investigation.

Patient 1 is a newborn female who was prenatally diagnosed with omphalocele and was noted to have a prominent tongue, thickened nuchal fold, short long bones, and a small right renal cyst. She presented at birth with omphalocele and respiratory distress. Noninvasive prenatal testing for the common trisomies (13, 18, and 21), monosomy X, and triploidy was low risk. She presented at birth with omphalocele and respiratory distress. On physical exam, she had macroglossia, overfolded superior helices, and creases and pits on the ear lobes. She was not hypoglycemic and had no simple nevi nor asymmetry. Microarray analysis detected a 419‐kb gain (duplication) in 11p15.5, with nomenclature arr[GRCh37] 11p15.5(2166707_2585400)x3 (Figure 1a).

**FIGURE 1:**
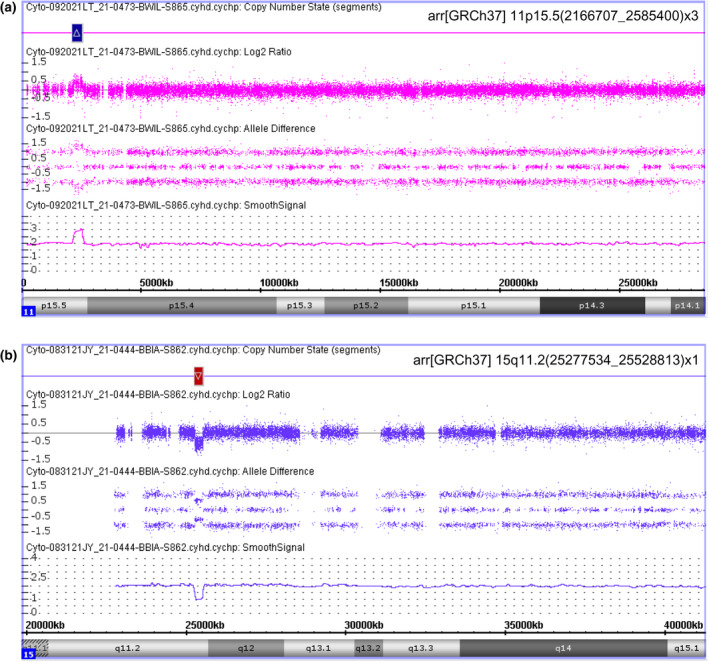
SNP microarray detection of copy number variations using the Affymetrix CytoScan HD platform. (a) Patient 1 showed a 419‐kb gain in chromosome 11. (b) Patient 2 showed a 251‐kb loss in chromosome 15

While there is no known triplosensitivity gene in this region, it is an imprinting region with imprinted genes clustered in two domains. The imprinting center 1 (*H19*/*IGF2*:IG DMR) and imprinting center 2 (*KCNQ1OT1*:TSS DMR) (Eggermann et al., 2015; Monk et al., 2018). Loss of methylation at the maternal imprinting center 2 has been found in many patients with Beckwith–Wiedemann syndrome (BWS), and hypomethylation of imprinting center 2 has also been reported (Eßinger et al., 2020; Valente et al., 2019). Interpretation of small duplications in this region requires the consideration of clinical features, together with genetic content, mode of inheritance, and methylation pattern (Heide et al., 2018).

Methylation studies for our patient, performed at ARUP, revealed normal methylation for the imprinting center 1 and hypomethylation for the imprinting center 2. These results, coupling with the finding of the 419 kb duplication involving the first two coding exons of the *KCNQ1*, supported the clinical diagnosis of BWS. Genetic counseling has been provided to the family. The recurrence risk would be assessed depending on whether the duplication is de novo or maternal in origin.

Patient 2 is a newborn male who was born at 38.2 weeks of pregnancy, with a birth weight of 3.5 kg, head circumference of 35.6 cm, and a length of 52.1 cm. Shortly after birth he had tachycardia for which echocardiogram was performed and showed patent foramen ovale and atrial septal defect. On physical exam, he was noted to have hypotonia, minimal primitive reflexes, and dysmorphic features which included low set ears, retrognathia, and deep nasal bridge. He also noted to have feeding difficulty. Microarray analysis detected a 251 kb loss (deletion) in 15q11.2, with nomenclature arr[GRCh37] 15q11.2(25277534_25528813)x1 (Figure 1b).

This deletion involved *SNHG14, SNORD116, IPW, PWAR1*, and *SNORD115*. Methylation study results, performed at LabCorp, were reported as “normal”. That report in the electronic medical record was understood by other providers to have ruled out the possibility of Prader–Willi syndrome (PWS). However, we interpreted the methylation results as “uninformative” because the methylation testing was performed using probes that were not in the deletion region. Our effort to secure methylation testing to evaluate the parental origin of the deletion was unsuccessful. To our knowledge, no diagnostic laboratory in the US currently offers methylation testing for this region.

PWS is an imprinting disorder involving chromosome 15q11‐q13. Paternal deletion of this region or maternal uniparental disomy of chromosome 15 is the major causes of PWS. Typical deletions in patients with PWS include the loss of *SNRPN*. This patient showed a smaller, atypical deletion including *SNHG14, SNORD116, IPW, PWAR1*, and *SNORD115*. This region, including *SNORD116*, a small nucleolar organizing RNA gene (previously named *HBII‐85*), has been recognized as a minimal critical region sufficient to cause multiple clinical features typical of PWS phenotype (Bieth et al., 2015; de Smith et al., 2009; Duker et al., 2010; Fontana et al., 2017; Sahoo et al., 2008).

Considering the possible inheritance nature of small CNVs, we recommended targeted microarray analysis of the affected region for both parents. A paternal inheritance would support the diagnosis of PWS and would be helpful for the recurrence risk assessment. Because of the phenotype and the detection of the loss of *SNORD116*, we provided genetic counseling to the family and offered monitoring of our patient as if he received a diagnosis of PWS.

It is worth noting that while methylation analysis is the preferred first‐tier testing for PWS (Ramsden et al., 2010), we have not been able to find a diagnostic laboratory that offers methylation study covering the *SNORD116* cluster region. We interpreted the methylation results as “uninformative” and hope to raise the awareness that microarray testing should be considered to detect small deletions in patients who show PWS features but have “normal” methylation results. We also hope that additional probes can be developed to cover the *SNORD116* cluster for methylation studies.

In summary, we report here the findings of small CNVs in two newborns in the genomic imprinting regions. They exemplified the challenge of interpreting small CNVs in diagnostic samples. Careful detection of small CNVs in the imprinting regions and effective genetic counseling are of clinical and reproductive significance. They can improve the diagnosis and management of the affected patients and their families.

## AUTHOR CONTRIBUTIONS

Carol Greene, Megan Czekalski, and Sylvia Herrada were responsible for acquisition of clinical information and genetic counseling. Yi Ning was responsible for microarray studies and drafted the manuscript. All authors have read and approved the final version of the manuscript.

## CONFLICT OF INTEREST

The authors declare no conflict of interest.

## Data Availability

Data sharing is not applicable to this article as no new data were created or analyzed in this study.
